# Subcapital Femoral Neck Tension Stress Fracture - A Rare Injury in A Child: A Case Report

**DOI:** 10.5704/MOJ.2103.021

**Published:** 2021-03

**Authors:** MF Hayyun, K Jamil, AH Abd-Rashid, S Ibrahim

**Affiliations:** Department of Orthopaedics and Traumatology, Universiti Kebangsaan Malaysia, Kuala Lumpur, Malaysia

**Keywords:** neck of femur, stress fracture, tension type, paediatrics

## Abstract

Femoral neck stress fractures are rare in children. To the best of our knowledge, the tension type stress fracture has been reported only twice in the English language literature. We report on a five years follow-up of a 10-year-old boy with this injury which was initially missed. The fracture healed after screw fixation. We highlight the importance of considering stress fracture as a differential diagnosis in a child with chronic hip pain. A careful physical examination and the appropriate imaging will avoid missing the diagnosis.

## Introduction

Stress fractures in skeletally immature patients have become more common due to the increased participation by children in sports. The tibia is the most frequently fractured bone but very few cases involving the neck of femur have been reported^1^. All reported cases of stress fracture of the femoral neck showed significant delay in diagnosis. Clinicians need to be aware of this condition when treating a child with long standing hip pain^2^. Devas in 1965 classified femoral neck stress fractures into compression and tension types. To the best of our knowledge, only two cases of tension type fracture in children have been published in the English language literature^1,3^.

## Case Report

**Fig. 1:**
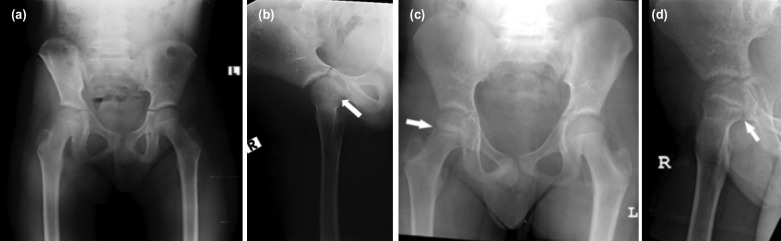
(a, b) Plain radiographs of the pelvis done immediately after the fall. The fracture was not obvious on the anterior-posterior radiograph but was visible on the lateral radiograph (arrow). (c, d) Plain radiographs five months after injury showing the posterolateral tension-type fracture in the right femoral neck (arrow).

A 10-year-old boy presented to our clinic with a five months history of limping and persistent pain in his right hip. The symptoms started after he fell while boarding a school bus. He was still able to weight bear despite the pain after the fall. He sought treatment at another hospital soon after the fall and had plain radiographs of the pelvis but the fracture was missed (Fig. 1a, 1b).

He was able to continue with his daily routine but was unable to undertake any strenuous activities. Oral analgesia only provided temporary pain relief. His parents sought a second opinion at our hospital as the child still had hip pain five months after the injury. Physical examination revealed an antalgic gait and reduced internal rotation of the right hip due to pain. Plain radiographs of the right hip showed a tension-type fracture the femoral neck (Fig. 1c, 1d).

**Fig. 2:**
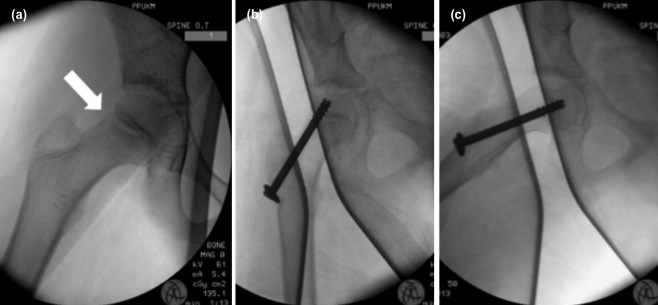
Intra-operative fluoroscopy images of the right hip. (a) Incomplete subcapital neck of femur fracture over the posterolateral aspect of the neck (arrow). (b, c) Antero-posterior and lateral views showing fixation with a single cannulated cancellous screw.

The child was admitted for an elective percutaneous screw fixation of the femoral neck. A single 16mm threaded 6.5mm cannulated cancellous screw was used to fix the fracture (Fig. 2).

**Fig. 3:**
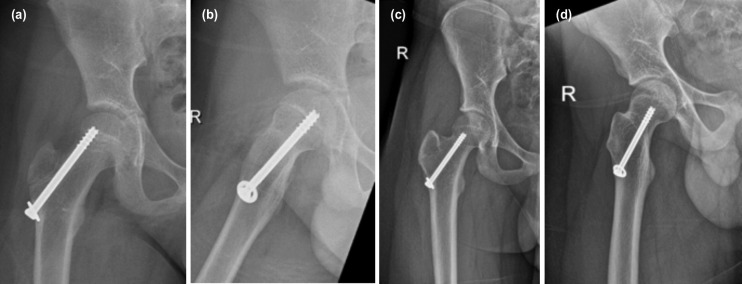
(a, b) Plain radiographs showing the healed fracture one year after surgery. (c, d) Plain radiographs five years after surgery showing a normal right hip.

He was pain free within a few days and was able to fully weightbear with crutches three weeks after surgery. The one-year follow-up radiograph showed the fracture had united. There was no evidence of avascular necrosis (Fig. 3a,b).

He subsequently defaulted follow-up for four years and was called back for a review. He remained asymptomatic. Although the screw fixation had crossed the growth plate, the plain radiograph at age 15 years did not show evidence of any growth arrest (Fig. 3c, d). The screw was subsequently removed.

## Discussion

Femoral neck fractures in children are rare and mainly caused by high impact trauma. Femoral neck stress fractures due to trivial trauma and repetitive movements are much rarer^4^. Devas in 1963 classified femoral neck stress fractures into compression and tension types^3^. Since then, several cases of femoral neck stress fractures have been reported but to the best of our knowledge, only two cases of femoral neck tension stress fractures have been reported^1,3^.

Femoral neck stress fractures may mimic the presentation of other painful hip pathologies such as Legg–Calve´–Perthes disease and slipped capital femoral epiphysis. In most reported cases, the fracture could be detected along the inner or outer trabeculae of the femoral neck or when new callus formation became visible.

In a child with chronic hip pain and restricted hip motion, further imaging studies are needed if the plain radiographs appear normal. Magnetic resonance imaging (MRI), computed tomography (CT) and bone scan are possible choices to be performed^2,4^. We did not use other imaging modalities as the fracture was clearly visible on a plain radiograph. In almost all reported cases, the fractures were successfully treated non-operatively with non or partial weight bearing^1,2,5^. Surgery is indicated for displaced fractures and tension type fractures^4^.

This fracture can be treated with a hip spica in younger children as they are unlikely to comply with non-weight bearing until fracture union^5^. The tension type fracture has a high tendency to progress into a complete fracture in adults and therefore warrants surgical intervention to ensure fracture healing.

We decided to treat our patient with screw fixation as he was 10-year-old and unsuitable for a hip spica. Screw fixation prevents joint stiffness and other cast related complications. Lehman and Shah treated a 14-year-old boy with a femoral neck tension stress fracture using two cannulated cancellous screws which did not cross the growth plate^3^. In contrast, we treated our patient with only a single cannulated cancellous screw as the fixation was stable. In spite of the screw crossing the growth plate, there was no evidence of growth arrest most likely due to the child approaching skeletal maturity.

Avascular necrosis of the head of femur is a well-known complication for displaced femoral neck fractures. The condition may develop as a result of occlusion of blood supply to the head of femur or the tamponade effect from increased intracapsular pressure from the fracture haematoma. The percentage of avascular necrosis in all types of neck of femur fracture was reported to be as high as 29%^4^. Avascular necrosis did not occur in our patient. The femoral neck stress fracture is a rare injury that can be missed in children. This injury must be considered in a child with chronic hip pain and restricted hip motion. Most cases can be detected from plain radiographs but further imaging will be needed if the plain radiographs appear normal. A young child may be treated in a hip spica while older children will require screw fixation.
